# P-1532. The Local Microbiota Modulates the Clostridioides difficile Metabolome to Promote Immunotolerance in the Colon: A potential Molecular Mechanism of Recurrent C. difficile Infection

**DOI:** 10.1093/ofid/ofaf695.1713

**Published:** 2026-01-11

**Authors:** Hadley Beauregard, Sean M Anderson, Cynthia L Sears, Abby Geis

**Affiliations:** Johns Hopkins, Baltimore, Maryland; Johns Hopkins Medicine, Baltimore, MD; Johns Hopkins University School of Medicine, Baltimore, Maryland; Johns Hopkins, Baltimore, Maryland

## Abstract

**Background:**

Of ∼500,000 *Clostridioides difficile* infections (CDI) reported annually, a particular challenge facing clinicians is the ∼30% of patients who face recurrent CDI (rCDI) following resolution of primary infection. Despite its widespread prevalence, the molecular and host-pathogen mechanisms that underscore the occurrence of rCDI remain relatively unknown.

**Methods:**

N/A

**Results:**

Methods and Results: To assess how members of the rCDI-associated microbiome affect *C. difficile (*Cd*)* pathogenicity, we infected BL/6 mice with Cd alone or by co-colonization with *Klebsiella pneumoniae* (Kp). Both groups displayed similar Cd gut colonization but mice co-inoculated with both bacteria had reduced infection severity, marked by higher body weight maintenance and less colon inflammation. To understand why Cd virulence was affected, we performed untargeted liquid-chromatography mass-spectrometry based metabolomics on cultures of Cd grown in the presence of gram-negative rod sterile supernatants, hypothesized to contain metabolites specific to the grown bacteria. Our results suggest that certain members of the microbiome induce a metabolic shift in Cd, defined by significantly increased production of the immunomodulatory tryptophan metabolite indole-3-acetic acid (IAA). Our results to date suggest that IAA significantly impacts Cd virulence by markedly increasing expression of *tcdB*, Cd’s primary virulence factor, and *sigE*, the master regulator of sporulation, 21-fold and 3-fold, respectively, relative to untreated cells (N=2 experiments). Further, our preliminary data support the hypothesis that treating mice with IAA during CDI lessens symptomatic infection while concurrently reducing clearance of Cd from the colon.

**Conclusion:**

Our results suggest that a metabolomic shift to IAA production in Cd is induced by a subset of microbial specific factors. We hypothesize that accumulation of colonic IAA promotes mucosal immunotolerance while simultaneously modifying pathogen virulence, which impacts the ability of the host to respond to and clear Cd from the mucosa. Understanding the molecular mechanisms and pathway(s) that induces IAA production may allow us to identify a biomarker in the stool of CDI patients who are at high-risk of recurrence.

**Disclosures:**

All Authors: No reported disclosures

